# Supplementary material for: A Novel Morphine Drinking Model of Opioid Dependence in Rats
Source: Int J Mol Sci. 2022 Mar 31;23(7):3874. doi: 10.3390/ijms23073874 (PMC8999131; doi:10.3390/ijms23073874)
Supplement: Supplementary file 1 [file ijms-23-03874-s001.zip › ijms-1629551-supplementary.pdf]

Supplementary Materials

A novel morphine drinking model of opioid dependence in rats

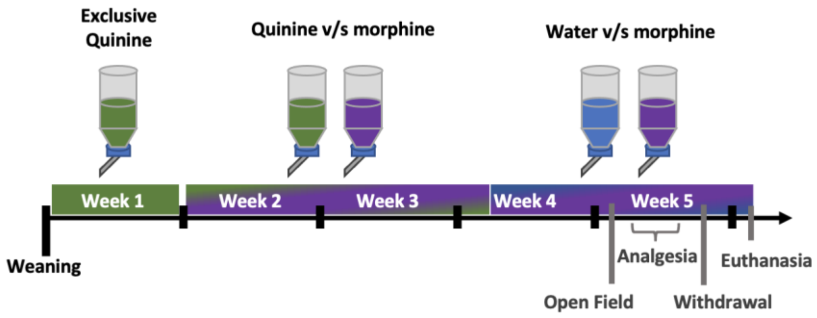

Supplementary Figure S1: Schedule of the oral consumption of experimental animals and behavioral tests**.**
